# A Case of a Grade 3 Gallbladder Neuroendocrine Tumor With Rapid Recurrence After Curative Resection

**DOI:** 10.7759/cureus.47193

**Published:** 2023-10-17

**Authors:** Naoki Iwanaga, Hiroyuki Sugo, Takuji Noro, Ikuo Watanobe, Kanako Ogura

**Affiliations:** 1 Department of General Surgery, Juntendo University Nerima Hospital, Tokyo, JPN; 2 Department of Diagnostic Pathology, Juntendo University Nerima Hospital, Tokyo, JPN

**Keywords:** neuroendocrine carcinoma(nec), bone metastasis, ki-67, rapid recurrence, gallbladder neuroendocrine tumor

## Abstract

Primary gallbladder neuroendocrine tumor (GB-NET) is extremely rare. Therefore, tumor behavior and adequate treatment in GB-NETs are still unclear. A 74-year-old man without any specific complaints was referred to our hospital cause of gallbladder tumor. Abdominal ultrasonography examination revealed a 22-mm non-pedunculated tumor in the gallbladder body. Contrast-enhanced computed tomography showed a polyp that was enhanced in the arterial phase. The patient underwent gallbladder bed resection and radical lymphadenectomy with a diagnosis of gallbladder carcinoma. Macroscopically, the resected specimen showed a nodular expanding tumor measuring 32×15 mm in the gallbladder body. From the pathological findings, a grade 3 GB-NET was diagnosed. Only cystic lymph node metastasis was observed. The patient was discharged uneventfully, but bone and lymph node metastasis were detected eight months after surgery. We conclude that grade 3 GB-NET shows occasionally malignant biological behavior although NET G3 is distinguished from neuroendocrine carcinoma in the current WHO 2019 classification of NET.

## Introduction

Primary gallbladder neuroendocrine tumors (GB-NET) are extremely rare, comprising a reported 0.5% of all NETs, and representing 2% of all gallbladder tumors [1]. Previously NET was called "carcinoid"; however, historically, this term has been associated with conflicting characteristics. In 1907, “karzinoide” (carcinoid) was described as a benign tumor by Oberndorfer [2]; however, such tumors were later suspected to be malignant because they have the ability to metastasize. In the last 20 years, the term carcinoid has been replaced by NET. The World Health Organization (WHO) proposed a new category in 2019, wherein GB-neuroendocrine neoplasms (GB-NENs) are divided into three categories, namely: NET (grade 1, 2, or 3 NETs), neuroendocrine carcinomas (NECs, large cell or small cell type), and mixed neuroendocrine-non-neuroendocrine neoplasms (MiNENs) [3]. However, such tumor behaviors and adequate treatment for this condition are still relatively unknown because this tumor is extremely rare [4,5].

We herein present an extremely rare case of a grade GB-NET in a 74-year-old man, which progressed to rapid bone and lymph node metastasis eight months after curative resection.

## Case presentation

A 74-year-old man without any specific complaints was referred to our hospital because of a gallbladder tumor that had been demonstrated by abdominal ultrasonography (US). His medical history included pneumothorax and gastric ulcer. The results of laboratory examinations, including liver function tests, and tumor markers such as CEA and CA19-9, were also within normal limits. 

**Figure 1:**
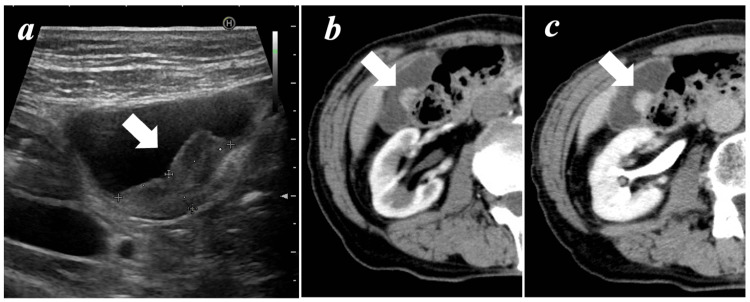
Abdominal ultrasonography (a); Abdominal tomography showing tumor in arterial phase (b) and venous phase (c) Abdominal ultrasonography revealed a well-defined, smooth mass of 22×8 mm inside the gallbladder (arrow) (a). Abdominal computed tomography showed a slightly enhanced tumor in the arterial phase (arrow) (b), however, there was no washout in the venous phase (c).

Abdominal US examination revealed a well-defined 22-mm non-pedunculated tumor in the gallbladder body (Figure 1a), indicating the presence of blood flow by color Doppler imaging. Neither gallbladder wall thickening nor gallbladder stones were found. Furthermore, enhanced computed tomography (CT) revealed an enhanced polypoid lesion in the arterial phase (Figure 1b), and no washout was observed in the venous phase (Figure 1c). 

**Figure 2:**
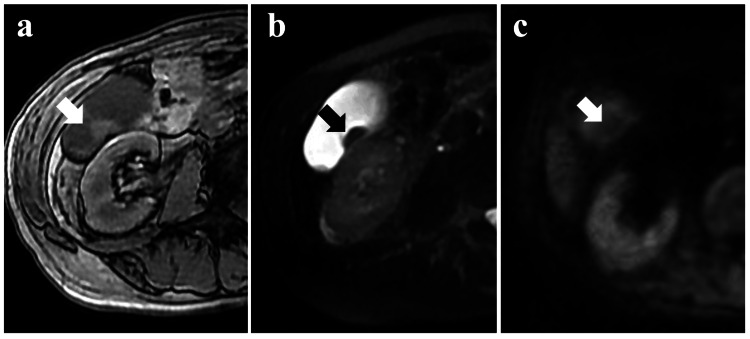
Abdominal magnetic resonance imaging The tumor shows iso-signal intensity on T1-weighted images (a) and low-signal intensity nodules on T2-weighted images (b). Low signal intensity is seen on diffusion-weighted images (c).

On magnetic resonance imaging, the lesion in GB showed slightly high signal intensity on T1-weighted images (Figure 2a), low signal intensity on T2-weighted images (Figure 2b), and slightly high signal intensity on diffusion-weighted images (Figure 2c). 

**Figure 3:**
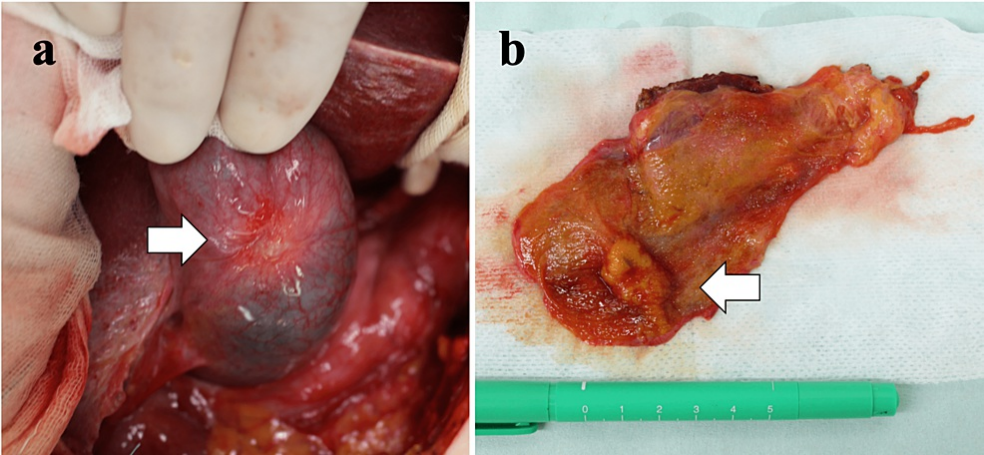
Intraoperative findings (a) and the resected specimen (b) Intraoperatively, shrinkage due to subserosal invasion is seen in the gallbladder body (arrow) (a). Macroscopically, the resected specimen showed a nodular expanding tumor measured 32×15 mm in the gallbladder body (arrow) (b).

The patient underwent gallbladder bed resection and radical lymphadenectomy under a diagnosis of gallbladder adenocarcinoma. Intraoperatively, shrinkage due to subserosal invasion was seen in the body of the gallbladder (Figure 3a) and cystic lymph node metastasis was also found. The swollen lymph nodes were easily dissected out, and the margins were clear. Macroscopically, the resected specimen showed a nodular expanding tumor measuring 32×15 mm in the gallbladder body (Figure 3b). 

**Figure 4:**
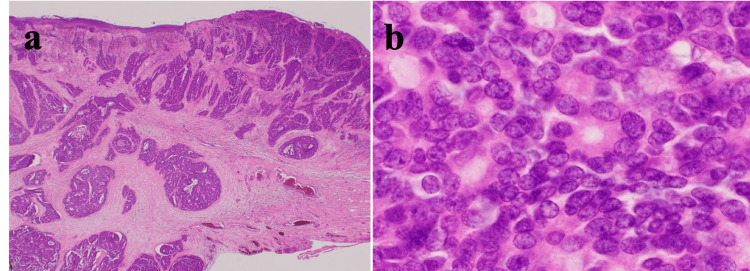
Histopathological findings Histologically, the tumor cells infiltrated diffusely through the gallbladder wall (a). The tumor cells had small round to oval nuclei with inconspicuous nucleoli, finely granular chromatin, and focal nuclear molding as well as rosette formation (b).

Immunohistochemical staining showed low nucleus-to-cytoplasm ratios exhibiting a fine granular chromatin pattern (salt-and-pepper appearance), which was suggestive of NET (Figure 4a-4b).

**Figure 5:**
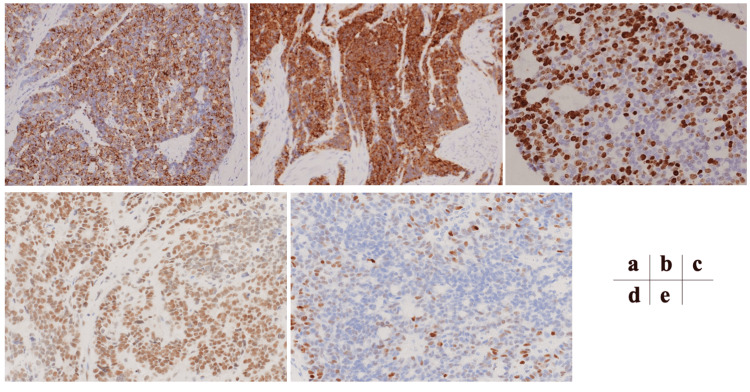
Immunohistochemical examination Immunohistochemical staining revealed positivity for chromogranin A (a; magnification, ×100) and synaptophysin (b; magnification, ×100). The Ki-67 index was 60% and the mitotic count was 25 per 10 high-power field (c; magnification, ×100). The tumor component was positive for ATRX (d; magnification, ×400), however, there was poor positivity for p53 (e; magnification, ×400).

Immunohistochemical staining revealed positivity for chromogranin A (Figure 5a) and synaptophysin (Figure 5b). Although the tumor showed moderate differentiation the marker of cellular proliferation Ki-67 index was high, approximately 60%. The mitotic count was 25 per 10 high-power fields (Figure 5c). Moreover, the tumor component was positive for ATRX and indicative of NEC rather than NET, however, it showed poor positivity for p53 with approximately 10%. According to the WHO 2019 classification, the tumor was diagnosed as a grade 3 NET. Only cystic lymph node metastasis was observed. Postoperatively, the patient was discharged on postoperative Day 10 without any complications. In the outpatient clinic, the patient was followed up without adjuvant chemotherapy, because conclusive evidence had not been established, especially in patients who underwent curative resection. However, multiple bone and lymph node metastases were detected eight months after the operation. Presently, 14 months from recurrence, the patient is alive and receiving combination chemotherapy with cisplatin and irinotecan. 

## Discussion

The mechanisms of occurrence of GB-NET are still unknown because neuroendocrine cells are not present in normal gallbladder mucosa. Previous reports have suggested that the progression of chronic inflammation induced gallbladder mucosal epithelial cells to deviate from their normal differentiation to develop an affected metaplastic epithelium, resulting in NEN. Thus, it was hypothesized that this was the reason for the frequent occurrence of coexisting gallstones and chronic cholecystitis in patients with GB-NET [6]. However, neither a history of cholecystitis nor gallbladder stones were found in the present case.

It is difficult to diagnose GB-NET preoperatively [6]. The diagnosis is made by radiological imaging only infrequently, because most patients have non-specific manifestations and the radiological features are similar to those of other tumors such as gallbladder cancer. GB-NETs are often diagnosed incidentally by routine pathological examination after cholecystectomy with a preoperative diagnosis of acute or chronic cholecystitis or with a gallbladder polyp. As NET can be one of several hypervascular gallbladder tumors, late arterial phase images should be obtained to differentiate NET from gallbladder cancer. For evaluation of lymph node metastases as well as distant metastases including liver metastases, portal venous phase radiography is also mandatory [7]. The present case highlights the imaging features of GB-NET, which can mimic the more common gallbladder carcinoma, but arterial hypervascularity and venous phase washout are suggestive of an alternative diagnosis [8].

In 2000, the WHO classification of digestive system tumors divided NETs into three subgroups: well-differentiated neuroendocrine tumors, well-differentiated neuroendocrine carcinomas, and poorly differentiated small-cell and large-cell neuroendocrine carcinomas. In 2010, the WHO updated the classification of NETs into three new categories according to the proliferative ability of the tumor: grade 1, 2, and 3 NETs [9]. In 2017, the WHO updated the classification of NET, as previously NEC had been subdivided into NET G3 and NEC (G3) [7]. According to the WHO (2019) classification system, GB-NENs are reclassified into four categories based on histological features: NET G1 (mitotic count <2/10 HPFs and/or Ki-67 index <3%), NET G2 (mitotic count = 2-20/10 HPFs and/or Ki-67 index = 3-20%), NET G3 (mitotic count >20/10 HPFs and/or Ki-67 index >20%), NEC (mitotic count >20/10 HPFs and/or Ki-67 index >20%), and MiNEN [3]. On the other hand, tumor behavior based on tumor grading has not been clarified because this tumor is extremely rare [4]. In the present case, the tumor showed the positivity of ATRX by immunostaining and was indicative of NEC rather than NET, however, p53 was negative. Consequently, the tumor was predominantly high-grade (grade 3 and undifferentiated), and thus diagnosed as NET G3. However, retrospectively, we suspected this tumor's characteristics were similar to NEC from such an aggressive course after surgery. If the tumor was suspected to be NEC, we thought that this case should be treated as NEC including aggressive adjuvant chemotherapy and close follow-up. On the other hand, the diagnosis of grading of NET can be sometimes difficult regardless of the immunohistochemical analysis available. In a study of pancreatic NET, Tang et al. suggested that additional practical modalities are required to facilitate an accurate diagnosis [10]. In 2017, pancreatic NECs were classified as NET G3 and NEC, and then NET G3 was newly adopted for the classification of gastrointestinal pancreatic neuroendocrine tumors in 2019. Thus, NET G3 is still rare and the disease frequency of NET G3 is still unknown. Wang et al. have reviewed 13 cases of GB-NET on the basis of the WHO 2019 classification and reported two cases as grade 1, two as NEC, and nine as MiNEN; no grade 3 case was found [11]. 

The adequate treatment strategy for GB-NET is not yet clarified. Complete en bloc surgical resection may be adequate as the only curative therapeutic treatment, especially in early-stage GB-NET. Moreover, the surgical procedure varies widely, ranging from a simple cholecystectomy to a radical hepatectomy with lymphadenectomy and hepatoduodenal ligament resection. Previous reports have demonstrated that tumor size is a risk factor for metastases in GB-NET, thus, NET G1 measuring less than 2 cm without lymph nodes and distant metastases have been treated by laparoscopic cholecystectomy as local excision [6,12]. On the other hand, in a case of clear cell GB-NET G1 measuring 0.8 cm in diameter, Hirose et al. demonstrated lymph node metastasis [13]. Yokoyama et al. have also reported that metastases were present in two (29%) of seven patients with tumors measuring less than 1 cm in diameter [14]. The risk factors for metastasis of GB-NET are not clear due to the paucity of reported cases. If lymph node metastasis was found, a radical resection with regional lymph node dissection should be required. In the present case, the tumor measured 32×15 mm, and the only cystic lymph node metastasis was observed intraoperatively; therefore, we performed radical liver resection and radical lymphadenectomy. However, rapid bone metastasis and lymph node metastasis developed eight months after surgery. Regarding with prognosis of GB-NET, Chen et al. demonstrated that GB-NEC according to the WHO 2010 classification had lymphatic metastases in 90% and their median survival time was 3.0 months, which is shorter than that for patients with gallbladder carcinoma [15]. From a review of biliary NENs according to the WHO 2010 classification, Zheng et al. demonstrated that NEC G3 showed a Ki-67 index as high as 60%, compared with 1% in G1 NET and 3% in G2 NET, and their recurrence rate was 54.2% [16]. The site of distant metastases was more frequent in the liver (91.7%), lungs (33.3%), lymph nodes (33.3%), bone (25%), and adrenal glands (25%) [6,12,13,16]. In the present case, a lymph node metastasis was observed and the Ki-67 index was 60%, as high as the NEC G3, even though the diagnosis was G3 NET. Obviously, tumor behaviors based on the WHO 2019 classification were not clarified, especially in grade 3 GB-NET. However, our patient might have a high risk for postoperative recurrence, as same as previous NEC G3. 

## Conclusions

Further studies to determine the prognostic factors of high-grade NETs such as G3 NET are needed in the future. The diagnosis of grading of NET was sometime difficult regardless the immunohistochemical analysis. We conclude that grade 3 GB-NET shows occasionally malignant biological behavior although NET G3 is divided from NEC in the WHO 2019 classification of NET. Accordingly, more aggressive treatment strategy and close follow-up would be necessary for patients with this rare tumor.
